# A Reply to Dr. T. F. Allen

**Published:** 1890-06

**Authors:** Charles B. Gilbert

**Affiliations:** Washington, D. C.


### A REPLY TO DR. T. F. ALLEN.

EDITORS HOMŒOPATHIC PHYSICIAN:—In answer to the fling at Dr. Hering made by Dr. T. F. Allen on p. 207 of the May number of your journal, I desire to say that Dr. Hering told me that he took up the Schüssler remedies because he saw their importance, and that he had the first edition published to keep them out of the hands of the mongrels.

He intended to have further provings made and to develop them as fast as possible; at the same time he was not averse to learning anything he could about the curative powers of medicines even if such knowledge had not been developed by provings on the healthy. It hardly becomes a physician—who, as Dean of an alleged homœopathic college, cries aloud through the journals to expectant students, "Come to our college! we teach you pure Homœopathy in one chair and false Homœopathy in the others; we will not let you go out of our halls without knowing both—to talk about Dr. Hering's indorsing Schüsslerism 'in a way;'" the "way" was by the publication spoken of, which did not indorse the *method* at all—only the remedies.

Learning the false, I suppose, must be useful to a homœopathist, on the ground that, knowing the devil, you can cut him when you meet him. Must virtuous people be familiar with sin before they can be properly virtuous? It is not whether a man *says* he is a homœopathist, it is whether he *is*. As between the purity of the Homœopathy of Dr. Hering, who in that very Schüssler pamphlet warns against pressing out the pus in gonorrhœa, because it injures the urethra, and that of Dr. Allen, who says that unless you are willing to be left behind your neighbor in the race, you must prescribe injections of mercuric chloride, I shall stand with Hering, although he was not a member of the New York County Homœopathic Society.

It is a mistake to call Dr. Allen ignorant; if so, there might be some excuse for his back-somersaults, which, somehow or other, all seem to have occurred since he became the mighty Dean who must run the machine in opposition to other—shops.

Truly yours,

CHAS. B. GILBERT, M. D.

WASHINGTON, D. C., April 29th, 1890.

